# The relationship between serum pro-B type natriuretic peptide level and bone mineral density in peritoneal dialysis patients

**DOI:** 10.1097/MD.0000000000034666

**Published:** 2023-09-22

**Authors:** Ayse Gulsen Doğan, Ulkem Uzeli, Baris Eser, Murat Dogan

**Affiliations:** a Physical Medicine and Rehabilitation Department, Hitit University Erol Olcok Training and Research Hospital, Corum, Turkey; b Department of Internal Medicine, Osmancik State Hospital, Corum, Turkey; c Department of Nephrology, Faculty of Medicine, Hitit University, Corum, Turkey; d Department of Internal Medicine, Faculty of Medicine, Hitit University, Corum, Turkey.

**Keywords:** B, bone mineral density, N, peritoneal dialysis, terminal pro, type natriuretic peptide

## Abstract

We aimed to evaluate the relationship between serum N-terminal pro-B-type natriuretic peptide (NT-pro-BNP) and lumbar bone mineral density (BMD) in peritoneal dialysis (PD) patients. Fasting blood samples were obtained from 46 PD patients. BMD was measured by dual-energy X-ray absorptiometry of the lumbar vertebrae (L1–L4). Circulating serum NT-pro-BNP levels were measured using commercial kits compatible with the Roche Cobas e 601 immunoassay device. Forty-six patients were included in our study. Increased age, low body mass index (BMI), and high-serum NT-pro-BNP are significantly associated with decreased BMD. The results show a statistically positive correlation between lumbar T-score values and BMI (r = 0.456; *P* = .001), while lumbar T-score values and PTH (rho = -0.336; *P* = .022) and log-NT-pro-BNP. There is a statistically negative correlation between BNP (rho = -0.355; *P* = .015). The lumbar T-score value decreases by 0.800 units when log-NT-pro-BNP increases by 1 unit and increases by 0.323 units when BMI increases by 1 unit. The established model is statistically significant (F = 6.190; *P* < .001). Our study in PD patients showed that serum NT-pro-BNP level was negatively correlated and BMI was positively correlated with lumbar BMD.

## 1. Introduction

Chronic kidney disease (CKD) is a structural or functional disorder of the kidney for 3 months or more.^[1]^ CKD is associated with the development of mineral bone disorder (MBD), osteoporosis, and fragility fractures.^[2]^ In patients with CKD, CKD-MBD develops in association with secondary hyperparathyroidism due to phosphorus (P) accumulation in the circulating plasma, leading to an increase in the risk of cardiovascular disease and bone fracture. It has been reported that the treatment/management of osteoporosis in CKD patients has a bearing on the progression of atherosclerosis, including vascular calcification, in that calcium (Ca) overload or hypercalcemia, coupled with the consequent suppression of bone turnover give rise to adynamic bone.^[3]^ N-terminal pro-B-type natriuretic peptide (NT-pro-BNP) is an inactive amino-terminal fragment, a cleavage product of pro-BNP, and in dialysis patients, elevated serum NT-pro-BNP is associated with hypervolemia and mortality.^[4,5]^ Various factors such as age, gender, activity of the renin-angiotensin system (RAS), and adipokine levels can affect bone mineral density (BMD). Natriuretic peptides are potent lipolytic agents that inhibit RAS and affect BMD.^[6,7]^ It has been clearly shown in the studies performed that there is a decreased BMD in hemodialysis and PD patients.^[8]^ However, there are few studies examining the relationship between NT-pro-BNP and BMD in dialysis patients. Hsu et al^[9]^ showed an inverse relationship between pro-BNP and bone density in renal transplant patients and Wang et al^[7]^ in peritoneal dialysis (PD) patients. The aim of this study is to examine the relationship of lumbar BMD results with serum NT-pro-BNP level in patients receiving PD and to identify a new biomarker to detect osteoporosis in PD patients.

## 2. Methods

This study was planned as a cross-sectional study. Forty-six PD patients at Hitit University Erol Olcok Training and Research Hospital were enrolled in March to December 2022. The study was approved by the Hitit University Clinical Research Ethics Committee (Date: May 25, 2022; No: 2022-51). A well-informed written consent was obtained from all participants according to the principles of the Declaration of Helsinki.

### 2.1. Patients

This group consisted of 27 men and 19 women (17 of whom were postmenopausal), and the age range was 20 to 81 years. The exclusion criteria were acute infection, malignancy, acute myocardial infarction, pulmonary edema, heart failure at the time of blood sampling, use of osteoporosis drugs (bisphosphonates, teriparatide, estrogen medications), and history of lumbar fracture or surgery.

#### 2.1.1. Anthropometric analysis

Body mass index (BMI) was calculated as the weight (kg) divided by height squared (m^2^).

### 2.2. Biochemical determinations

Routine biochemical analyzes were performed in the central laboratory of our hospital. Fasting blood sugar, total cholesterol (TK), triglyceride (TG), high-density lipoprotein cholesterol (HDL-C) and low-density lipoprotein cholesterol (LDL-C), serum creatinine, calcium, alkaline phosphatase (ALP), hemoglobin (Hb), white blood cell, neutrophil and lymphocyte counts, neutrophil/lymphocyte ratio (NLO), 25 hydroxyvitamin-D (25-OH-D), and circulating BNP values were recorded. Circulating serum NT-pro-BNP levels were measured using commercial kits (Elecsys proBNPII) compatible with the Roche Cobas e 601 immunoassay device (Roche Diagnostics Australia Pty Limited Australia). The normal range was 0 to 125 pg/mL.

### 2.3. BMD measurement and diagnosis of osteoporosis

Measurement of the hip and spine with dual-energy X-ray absorptiometry (DEXA) is used in today’s technology for the prediction and follow-up of a fracture that may occur in the future. BMD is the expression in grams (g/cm^2^) of the mineral per square centimeter of a certain area of the bone. The result is evaluated by establishing a connection with 2 different criteria. As a standard, the T-score is used in the measurement results, which is the comparison of the bone density of young healthy people of the same race and sex with the bone density of the patient. The Z-score, on the other hand, compares the patient with individuals of the same age group but of the same race and sex. According to the World Health Organization diagnostic criteria, if the BMD T-score is >-1, it is classified as normal, between -1 and -2.5 as osteopenia, ≤-2.5 as osteoporosis, and ≤-2.5 and fragility fracture as severe osteoporosis.^[9]^

#### 2.3.1. Sample size

The power analysis result based on the NT-pro-BNP value obtained from the study of Wang et al^[7]^ revealed that type I error (alpha) was 5% (*P* =.05) and type II error (beta) was 0.2% (80%). The sample size was calculated as 35 patients, assuming that the we included 46 patients in our study.

### 2.4. Statistical analysis

Analyzes were made with the SPSS 23.0 program. All data were first analyzed for normal distribution using the Kolmogorov-Smirnov test, and then Levene test was used to test the assumption of homogeneity of variance. Data were expressed as mean ± standard deviation for continuous variables or numbers (percentages) for categorical variables. Differences between 3 or more groups were evaluated using 1-way analysis of variance (ANOVA) (continuous variables with normal distribution and homogeneity of variance) or the Kruskal-Wallis test (variables with nonparametric distribution and/or variance). Correlation analysis was used to evaluate the relationship between circulating BNP and other variables. The associations of circulating BNP and other variables with osteoporosis risk were performed using univariate and multivariate logistic regression analyses. Odds ratios and 95% confidence intervals were estimated. ROC curve analysis was performed to determine the optimal cutoff point of circulating BNP for the diagnosis of osteoporosis. *P* < .05 was considered statistically significant.

## 3. Results

Forty-six patients were included in our study. The ages of the patients in the study ranged from 20 to 81 years, with a mean age of 59.45 ± 13.58 years. Of the patients, 27 (58.7%) are male, 28 (60.9%) and 39 (84.8%) were married. The number of smokers is 6 (13.0%), and the number of patients using alcohol is 1 (2.2%). DM is present in 41 (89.1%) patients, and HT is present in 22 (47.8%) patients. The number of patients using insulin is 20 (43.5%), and the number of patients using benidipine is 20 (43.5%). The results in Table 1 show that increased age, low BMI, and high-serum NT-pro-BNP are significantly associated with decreased BMD. The results in Table 2 show a statistically positive correlation between lumbar T-score values and BMI (r = 0.456; *P* = .001), while lumbar T-score values and PTH (rho = -0.336; *P* = .022) and log-NT-pro-BNP. There is a statistically negative correlation between BNP (rho = -0.355; *P* = .015). In Table 3, the effects of age, BMI, and NT-pro-BNP on lumbar T-score values were evaluated by linear regression analysis. According to the stepwise method, log-NT-pro-BNP and BMI were found to be statistically significant in the final model. According to Table 3, the lumbar T-score value decreases by 0.800 units when log-NT-pro-BNP increases by 1 unit, and increases by 0.323 units when BMI increases by 1 unit. The established model is statistically significant (F = 6.190; *P* < .001). Variables in the model explain the lumbar T-score value at 36.6%.

**Table 1:** Clinical features of 46 peritoneal dialysis patients according to T-scores.

	Osteopenia (n = 18) –2.5 < T-score ≤ –1	Osteoporosis (n = 13) T-score ≤ –2.5	Normal (n = 15) T-score > –1	*P*
Age (year)	59.72 ± 10.03	59.69 ± 17.11	48.93 ± 13.59	**0.004** †
BMI (kg/m^2^)	27.62 ± 4.68^a^	25.67 ± 5.46^a^	32.67 ± 8.49^b^	**0.016** †
Peritoneal dialysis duration (month)	47.06 ± 37.55	38.01 ± 34.66	40.99 ± 39.01	0.698†
Lumbar T-score	-1.51 ± 0.43^a^	-2.70 ± 0.76^b^	0.70 ± 1.04^c^	**<0.001** †
Lumbar Z-score	-0.57 ± 0.71^a^	-1.88 ± 1.38^b^	1.50 ± 1.32^c^	**<0.001** †
Systolic blood pressure (mm Hg)	139.16 ± 20.45	139.23 ± 15.52	131.33 ± 21.33	0.447†
Diastolic blood pressure (mm Hg)	76.94 ± 14.05	77.69 ± 9.26	75.33 ± 10.30	0.861†
Fasting glucose (mg/dL)	144.00 (118.50)	118.00 (186.00)	184.00 (125.00)	0.547‡
Uric acid	5.43 ± 1.53	5.23 ± 1.03	6.10 ± 1.06	0.177†
Creatinine (mg/dL)	6.35 ± 1.92	5.55 ± 2.98	6.96 ± 2.15	0.294†
Total protein	66.00 (11.25)	67.00 (9.50)	67.00 (8.00)	0.702‡
Albumin (g/dL)	33.11 ± 3.77	32.15 ± 5.09	33.73 ± 4.94	0.660†
Phosphorus (mg/dL)	4.25 ± 0.77	4.30 ± 1.04	4.62 ± 1.20	0.545†
Total Calcium (mg/dL)	8.66 ± 1.35	8.84 ± 0.72	8.95 ± 0.87	0.727†
Triglyceride (mg/dL)	122.50 (107.50)	181.00 (87.50)	156.00 (89.00)	0.814‡
Total cholesterol (mg/dL)	163.00 (76.00)	181.00 (29.00)	179.00 (58.00)	0.355‡
HDL-C (mg/dL)	44.50 (21.00)	45.00 (21.00)	49.00 (19.00)	0.815‡
LDL-C (mg/dL)	87.00 (56.50)	102.50 (35.25)	96.00 (34.00)	0.371‡
Intact parathyroid hormone (pg/mL)	268.50 (382.50)	309.00 (224.00)	198.00 (149.00)	0.285‡
NT-pro-BNP	5394.00 (6153.75)	5934.00 (6622.00)	2967.00 (4922.00)	**0.002**‡

Summary statistics for numerical variables are given as mean ± standard deviation.

*More than 1 option could be ticked.

† One-way analysis of variance.

γChi-square test based on Exact method.

a

b show the difference between groups.

There is no statistical difference between groups with the same superscripts. Significance value written as bold is statistically significant at *P* < .05 level.

BMI = body mass index, CAD = coronary artery disease, CHF = chronic heart failure, DM = diabetes mellitus, HDL-C = high-density lipoprotein cholesterol, HPL = hyperlipidemia, HT = hypertension, LDL-C = low-density lipoprotein cholesterol, NT-pro-BNP = N-terminal pro-B-type natriuretic peptide.

**Table 2:** Correlations between lumbar T-score and clinical variables.

	Lumbar T-score	
	*r*	*P*
Age	0.048	0.753
BMI	**0.456**	**0.001**
Log-NT-pro-BNP	**-0.355**	**0.015**
	rho	*P*
PTH	**-0.336**	**0.022**

r: Pearson correlation coefficient, rho: Spearman correlation coefficient, those written in bold are statistically significant at *P*<0.05.

BMI = body mass index, NT-pro-BNP = N-terminal pro-B-type natriuretic peptide.

**Table 3:** Multivariate stepwise linear regression analysis of the association of age, body mass index, and log-NT-pro-BNP with lumbar bone mineral density in 46 peritoneal dialysis patients.

	*β*	*sh*	*zβ*	*t*	*P*	95% confidence interval for β	
						Lower limit	Upper limit
**Log-NT-Pro-BNP**	-0.800	0.378	-0.262	-2.116	0.041	-1.564	-0.036
**BMI**	0.323	0.149	0.265	2.162	0.037	0.021	0.624

β: Regression coefficient, sh: standard error, zβ: Standardized regression coefficient, Model Statistics: F = 6.190; *P* < .001, R2 = 0.436, adjusted R2 = 0.366, multiple linearity statistics: tolerance = 0.888–0.951; variance inflation factor = 1.029–1.126.

BMI = body mass index, NT-pro-BNP = N-terminal pro-B-type natriuretic peptide.

## 4. Discussion

As a result of this study, it was concluded that there is a significant and independent positive relationship between BMI and lumbar BMD, and NT-pro-BNP has a significant and independent negative relationship with lumbar BMD. In addition to these results, increased NT-pro-BNP values in the 3 BMD groups were significantly associated with a lower lumbar T-score.

CKD is a systemic disease and affects many systems throughout its clinical course. The most important cause of mortality and morbidity in CKD is cardiovascular diseases, and bone mineral disorders also contribute to increased mortality. It provides this contribution both by increasing the risk of bone fracture and by increasing the risk of cardiovascular disease. This disorder is defined as CKD-MBD.^[10,11]^ Another MBD in CKD is osteoporosis. Osteoporosis is a structural failure of bone characterized by low bone mass and deterioration of the microarchitectural structure of bone tissue. Due to the deterioration of the bone structure, the bone becomes fragile and becomes easier to break. In a study published by Dusceac et al^[12]^ in 2018, people with normal kidney function and hemodialysis patients were compared, and it was concluded that trabecular bone density in the lumbar vertebrae was lower in hemodialysis patients and the 10-year fracture risk was higher. In the study of Jadoul et al^[13]^ in PD patients, osteoporosis (15.4%) and osteopenia (38.5%) of the femoral neck, in the study of Jeoung et al,^[14]^ osteoporosis of the femoral neck (7%) and lumbar spine (17%), and osteoporosis of the femoral neck (53%) and lumbar spine osteopenia in the spine (41%), and in the study of Wang et al,^[7]^ 19.2% had osteoporosis and 44.2% had osteopenia in the lumbar spine. In a study by Aslan et al,^[15]^ osteoporosis was detected in 32 (43%) of 74 patients, and osteoporosis was found in 13 (35.1%) patients using PD and 19 (51.4%) patients using hemodialysis. In our study, 28.2% of PD patients had osteoporosis and 39.1% had osteopenia of the lumbar spine.

Low BMI and low body weight is a known risk factor for osteoporosis.^[16]^ Studies have found that osteoporosis is observed more frequently in patients with low body weight and low BMI, who underwent peritoneal and hemodialysis.^[15,17,18]^ A positive correlation was also shown between BMI and BMD in renal transplantation patients.^[9,19]^ In our study, a positive correlation was found between BMI and BMD.

The natriuretic peptides, namely brain natriuretic peptide (BNP) or N-terminal pro-brain natriuretic peptide (NT-pro-BNP), belong to a family of vasopeptide hormones that are released from the heart and play a major role in blood pressure regulation and volume homeostasis through their direct effects on the kidney and systemic vasculature and represent a favorable aspect of neurohumoral activation.^[20]^

In CKD patients receiving either hemodialysis or PD treatment, BNP and NT-pro-BNP are frequently elevated compared with the normal cutoff values. In dialysis patients, elevated serum NT-pro-BNP is associated with hypervolemia and mortality.^[21]^ BNP plays a regulatory role against increased ventricular volume load, vasoconstriction, sodium retention, and RAS activation.^[22,23]^ Natriuretic peptides can inhibit RAS, leading to decreased BMD. Wang et al^[7]^ study conducted by 52 PD patients, a significant correlation was found between increased serum NT-pro-BNP value and low BMD. Lee et al^[24]^ study on 69 renal transplantation patients, a negative correlation was found between serum NT-pro-BNP value and lumbar T-score. In addition, Kajita et al^[25]^ performed quantitative locus analysis on postmenopausal patients and showed that BNP variation may be an important determinant of postmenopausal osteoporosis because it causes bone loss mechanism. In studies conducted in T2DM patients, a negative correlation was found between circulating BNP and BMD.^[26]^ In our study, we found that increased serum NT-pro-BNP levels were associated with decreased lumbar T-scores, and serum levels of NT-pro-BNP were an important and independent predictor of lumbar BMD in PD patients.

### 4.1. Limitation

The limited number of patients and the absence of a control group are limitations of this study. No control group was used, as it was planned as a cross-sectional study. Studies involving more patients are needed to use NT-pro-BNP as a marker of osteoporosis in dialysis patients.

## 5. Conclusions

In conclusion, our study in PD patients showed that serum NT-pro-BNP level was negatively correlated and BMI was positively correlated with lumbar BMD. Studies involving more patients are needed to use NT-pro-BNP as a marker of osteoporosis in dialysis patients.

## Author contribution

**Conceptualization:** A.G.D.

**Data curation:** A.G.D.

**Formal analysis**: A.G.D. and Ü.Ş.U.

**Investigation:** A.G.D., Ü.Ş.U., M.D., and B.E.

**Methodology:** A.G.D., Ü.Ş.U., M.D., and B.E.

**Project administration:** A.G.D.

**Resources:** A.G.D., Ü.Ş.U., M.D., and B.E.

**Software:** A.G.D., Ü.Ş.U., M.D., and B.E.

**Supervision:** A.G.D., Ü.Ş.U., M.D., and B.E.

**Validation:** A.G.D., Ü.Ş.U., M.D., and B.E.

**Visualization:** A.G.D.

**Writing—original draft**: A.G.D., Ü.Ş.U., M.D., and B.E.

**Writing—review and editing**: A.G.D., Ü.Ş.U., M.D., and B.E.

**Formal analysis:** U.Ş.U. and B.E.

**Funding acquisition:** U.Ş.U.
